# Agricultural or gardening physical activity may slow neurovascular aging and prevent stroke and dementia: an experimental and cross-sectional study

**DOI:** 10.3389/fnagi.2025.1676259

**Published:** 2025-12-02

**Authors:** Kiyoshi Kikuchi, Seiya Takada, Shotaro Otsuka, Kazuki Nakanishi, Harutoshi Sakakima, Hyuma Makizako, Nobuhiro Tahara, Hisaaki Uchikado, Naoto Shiomi, Satomi Ooba, Naoyuki Matsumoto, Masato Nishiwaki

**Affiliations:** 1 Division of Brain Science, Department of Physiology, Kurume University School of Medicine, Kurume, Japan; 2 Department of Neurosurgery, Kurume University School of Medicine, Kurume, Japan; 3 Department of Orthopedic Surgery, Graduate School of Medical and Dental Sciences, Kagoshima University, Kagoshima, Japan; 4 Laboratory for Statistical and Translational Genetics, RIKEN Center for Integrative Medical Sciences, Yokohama, Japan; 5 Faculty of Welfare and Health Sciences, Oita University, Oita, Japan; 6 Department of Physical Therapy, School of Health Sciences, Faculty of Medicine, Kagoshima University, Kagoshima, Japan; 7 Division of Cardiovascular Medicine, Kurume University School of Medicine, Kurume, Japan; 8 Uchikado Neuro-Spine Clinic, Fukuoka, Japan; 9 Department of Critical and Intensive Care Medicine, Shiga University of Medical Science, Otsu, Japan; 10 Department of Neurosurgery and Headache, Ooba Clinic, Oita, Japan; 11 Faculty of Environmental Symbiotic Sciences, Prefectural University of Kumamoto, Kumamoto, Japan; 12 Faculty of Engineering, Osaka Institute of Technology, Osaka, Japan

**Keywords:** arterial stiffness, brain-derived neurotrophic factor, cerebral white matter hyperintensities, dementia, magnetic resonance imaging, plasmin-α2-plasmin inhibitor complexes, simulated agricultural or gardening physical activity, stroke

## Abstract

**Background:**

Agricultural or gardening activity (also known as hobby farming) is a simple strategy that may be effective for maintaining health and preventing lifestyle-related diseases. However, its preventive effect on the development of conditions associated with neurovascular aging (e.g., stroke and dementia) remains unclear.

**Objective:**

To comprehensively investigate the preventive role of regular agricultural or gardening physical activity (AGPA) in neurovascular aging and its underlying mechanisms using two approaches.

**Methods:**

We conducted an experimental study in which we assessed arterial stiffness, cognitive performance (Flanker and Stroop tests), and circulating biomarkers (e.g., plasmin-α2-plasmin inhibitor complexes, nitric oxide, brain-derived neurotrophic factor) in 12 male students (average age: 22 ± 1 years) before and after three 40-min interventions (resting, cycling, and simulated AGPA) under controlled conditions. We also conducted a cross-sectional study, in which we recruited 161 (79 in the AGPA group and 82 in the control group) hospital-based older individuals (average age: 78 ± 5 years) and assessed their history of stroke, cognitive function, and brain magnetic resonance imaging (MRI) findings.

**Results:**

In the experimental study, simulated AGPA reduced arterial stiffness, improved executive cognitive function, and elevated circulating plasmin-α2-plasmin inhibitor complexes, nitric oxide, and brain-derived neurotrophic factor. Brain MRI-assessed cerebral white matter hyperintensities caused by reduced blood flow to brain tissue and stroke prevalence were lower, and cognitive scores (as assessed by the Hasegawa Dementia Scale-Revised) were higher in the AGPA group than in the control group.

**Conclusion:**

Our findings suggest that regular AGPA is associated with markers of slower neurovascular aging in older individuals. AGPA induces a combination of general physical activity-related and specific AGPA-related effects; moreover, it may offer similar or even greater benefits than physical activity alone. Therefore, habitual AGPA may serve as an effective preventive strategy for neurovascular aging.

## 1 Introduction

Epidemiological observational studies have suggested that farming is one of the healthiest occupations. A study conducted in Australia showed that farmers are a third less likely to suffer from a chronic illness, and 40% less likely to visit a general practitioner than non-farm workers (Brew et al., 2016). Researchers from the United States compared mortality rates between farmers and the general population and found that farmers are less likely to die from cancer, heart disease, and diabetes (Waggoner et al., 2011). Furthermore, several studies conducted in Western countries have revealed that farmers are healthier than non-farmers (Blair et al., 1992; Fleming et al., 1999; Stiernström et al., 2001; Blair et al., 2005; Waggoner et al., 2011; Armitage et al., 2012; Levêque-Morlais et al., 2015).

In Japan, farmers similarly have a longer lifespan than non-farm workers. Moreover, in terms of cause of death, mortality rates for senility and cardiovascular disease are particularly low in farmers (Kawasaki, 2015). The Japan Institute of Rural Medicine reported that farmwork has a positive impact on the physical functions of older adults. For example, farmwork has been shown to prevent chronic illnesses such as diabetes and hyperlipidemia (Matsumori et al., 2009). Because Japan has an extremely small land area and an extremely high population density, the Japanese agricultural industry comprises many small family farms, unlike Western countries, which run large-scale corporate operations. In Japan, approximately two-thirds of farming households are part-time or older adult retirees, and only a small fraction are full-time self-employed farmers (Zollet and Maharjan, 2021). Therefore, it is plausible that even light farming activities (including gardening or hobby farming) contribute to good health. Indeed, Buettner reported that many centenarians globally share one common hobby: gardening (Buettner and Skemp, 2016). Willcox’s observation of centenarians living in Okinawa, Japan, revealed that many residents maintain small personal gardens well into older adulthood (Jamie Feldmar, 2018). Additionally, Breen et al. reported that public health advice on exercise training for older adults is often quite vague, and that activities such as gardening started later in life can improve overall health if undertaken as part of a regular physical activity regime (Stephenson, 2019). Furthermore, high-intensity exercise may not be necessary for older adults to maintain health, and even gardening can reduce the risk of stroke and coronary heart disease (LaCroix et al., 2019; National Institutes of Health, 2019). In line with these findings, a meta-analysis of research examining the effects of gardening and horticultural therapy on health reported a wide range of positive health outcomes, such as reductions in depression, anxiety, and body mass index, as well as increases in life satisfaction, quality of life, and a sense of community (Soga et al., 2016).

Despite these findings, it remains unclear whether the factors that contribute to the good health of farmers and gardeners are related to physical activity, the psychological effects of engaging with nature (Smith et al., 2019; Watanabe et al., 2021), or their diet. Observational findings, statistical reports, and subjective indices alone are insufficient for assessing whether farming- and gardening-related physical activity [herein referred to as agricultural or gardening physical activity (AGPA)], such as hand weeding, digging, pruning, mixing soil, filling containers with soil, fertilizing, raking, planting, staking plants, sowing seeds, mulching, watering, harvesting produce, and washing produce (Nicklett et al., 2016), induces favorable effects, and a more detailed and comprehensive study is needed. Therefore, this study aimed to determine the factors that contribute to the good health of farmers and gardeners, with a focus on physical activity.

To the best of our knowledge, there have been no reports on the specific physiological mechanisms by which AGPA contributes to good health, especially the prevention of conditions related to neurovascular aging, such as stroke and dementia (Graham, 2017). First, we conducted an experimental study to determine the effects of farmwork on neurovascular aging by examining blood vessels. Additionally, we investigated the effects on vascular function using simulated indoor AGPA (sAGPA) instead of actual outdoor farmwork.

There have also been no reports using magnetic resonance imaging (MRI) data to investigate whether AGPA has an impact on the development of stroke or dementia in those who participate in gardening, retirees, and part-time and full-time self-employed farmers. Globally, the 10 most common causes of death include two diseases related to neurovascular aging: stroke and dementia (World Health Organization, 2024). In Japan, the top two leading causes of death in 2021 were dementia and stroke (GBD 2021 Japan Collaborators, 2025). In aging societies such as Japan, dementia is the leading cause of requiring long-term care, followed by stroke (Ministry of Health, Labour and Welfare, Patient Survey, 2020). Individuals with farm work experience had a shorter late-life dependency duration than those without farm work experience (Haruyama et al., 2020). Demonstrating that AGPA has a positive effect on both cognition and brain structure is crucial evidence that such activities are beneficial. Therefore, we used brain MRI data from patients to obtain important health management information about older adults. Understanding lifestyle habits across the lifespan is important for developing ways to sustain healthy neurovascular aging (Piercy et al., 2018). Given the “super-aging society” of Japan, our data offer valuable lessons for other countries worldwide in anticipation of future challenges. In an aging world with ongoing, urgent environmental concerns, our primary aim was to identify lifestyles or hobbies that individuals can easily implement to promote both human and environmental health on a local scale. To this end, we examined whether AGPA has a beneficial effect on arterial stiffness, cerebral white matter hyperintensities (WMHs), the development of age-related neurovascular disorders (Huang et al., 2022), and cognitive function, thereby preventing stroke and dementia.

## 2 Materials and methods

### 2.1 Ethics approval and consent to participate

**Figure 1:**
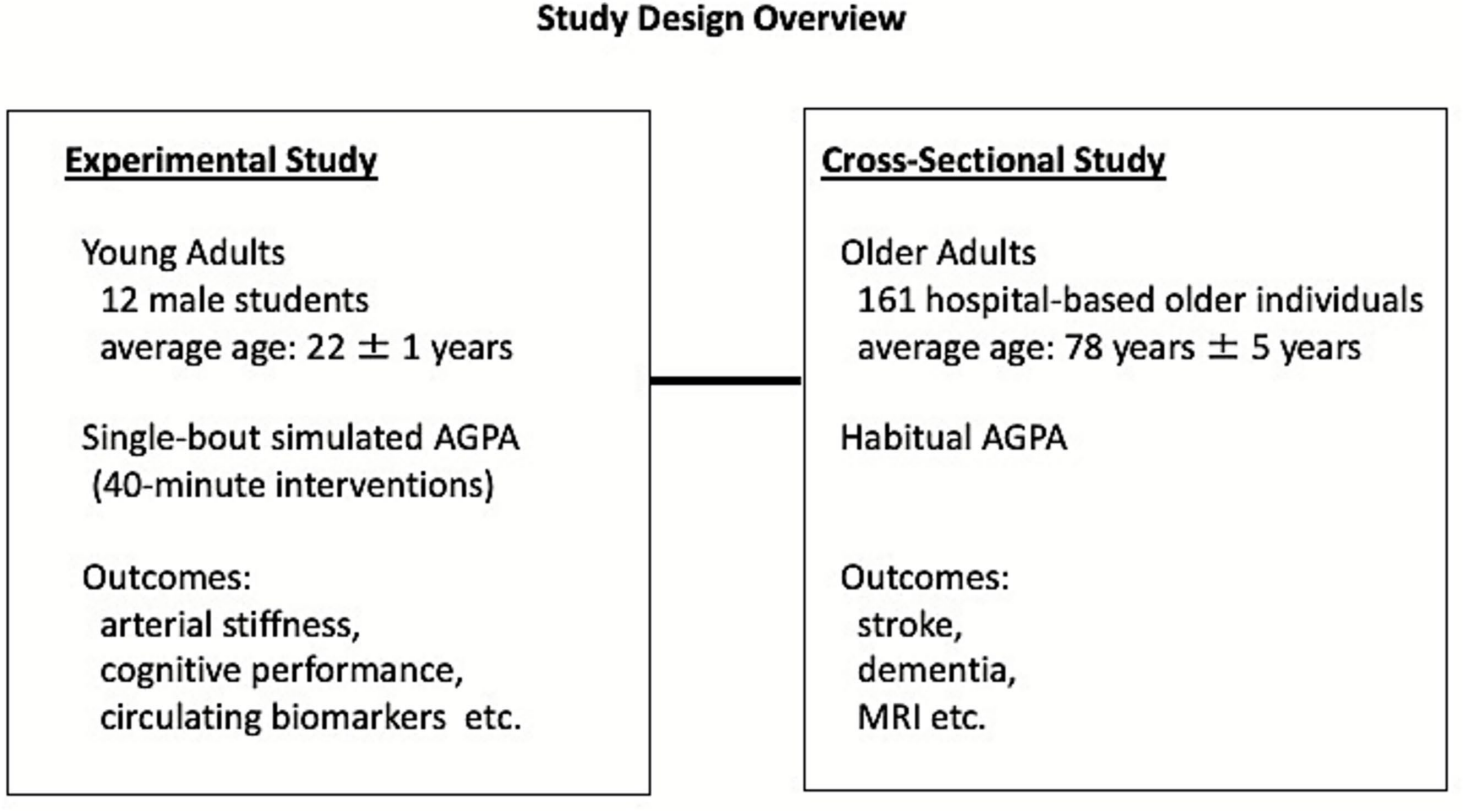
Study design overview. We conducted two different studies to evaluate the effects of agricultural or gardening physical activity (AGPA): an experimental study in young adults and a cross-sectional study in older adults.

We conducted an experimental study in young adults and a cross-sectional study in older adults (Figure 1). The experimental study was approved by the Human Ethics Committee of the Osaka Institute of Technology (2019–13), and the cross-sectional study was approved by the Ethics Committee of Miyazaki Hospital (30/349). The procedures used in this study adhere to the tenets of the Declaration of Helsinki. Written informed consent was obtained from all participants before study enrollment.

### 2.2 Experimental study for healthy adults using sAGPA

#### 2.2.1 Objectives

We aimed to investigate whether AGPA alters arterial stiffness, cognitive function, and circulatory parameters, and determine the factors involved in AGPA-induced preventive effects, other than those related to physiological and psychological effects of exposure to the natural environment (e.g., exposure to daylight and outside air/winds), by assessing sAGPA-induced typical physiological responses in healthy adults. In this study, sAGPA involved completing mock gardening activities in a laboratory-based artificial garden space (see section 2.2.3 for details).

#### 2.2.2 Participants

The inclusion criteria were healthy, young adults (aged ≤ 25 years). We recruited healthy Asian college students who were attending Osaka Institute of Technology via local advertisements and referrals from July 2019 to August 2019. Twelve male students agreed to participate in the study; there were no female respondents, which may be attributed to the predominantly male population at the college. Age, height, weight, and body mass index (BMI) of participants were 22 ± 1 years, 169.9 ± 5.0 cm, 62.7 ± 7.6 kg, and 21.7 ± 2.3 kg/m^2^, respectively. Age was self-reported, whereas we measured height, weight, and BMI. None of the participants had a chronic disease that could affect cardiovascular health, metabolism, or daily physical activity, or a history of smoking, and none were taking medication.

Before the study, appropriate and minimum sample sizes were calculated using G*Power version 3.1 (Heinrich Heine University, Düsseldorf, Germany). To detect an effect size (f) of 0.25 (medium) and an effect size (dz) of 1.0 (a difference of ≥ 7% arterial stiffness) at 80% power with an *α* of 5% using a within-between interaction of two-way repeated-measures analysis of variance (ANOVA) and a simple main effect of the pretest-posttest design, we planned to recruit 12 participants (12 participants × each 3 trials = total sample size: 36).

#### 2.2.3 Experimental procedures

**Figure 2:**
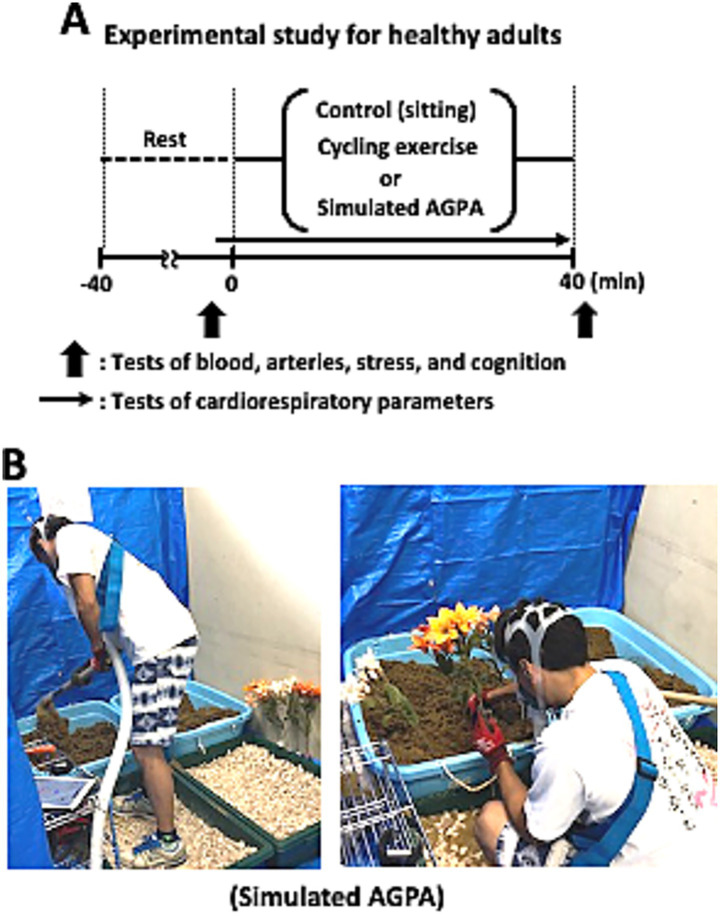
Time course of the experiment and example image of the experimental study using simulated agricultural or gardening physical activity (AGPA) in healthy adults. To explore the preventive effects of AGPA on neurovascular aging, we used experimental study data. **(A)** Over 3 days, 12 adults completed the three conditions of control, cycling exercise, and simulated AGPA in random order. **(B)** Example image of the simulated AGPA trial. AGPA, agricultural or gardening physical activity.

All trials were performed at the same time of day, at least 4 h after a light meal, in a quiet, air-conditioned (22–24 °C) laboratory room at Osaka Institute of Technology, Japan, in August 2019. The same researcher performed all of the experimental interventions and physiological outcome measurements. Participants were advised to eat the same meals (i.e., breakfast, lunch, and dinner) the day before each experimental session. Each participant completed three 40-min trials separately in random order at one trial per day for 3 days. The three trials comprised: (1) resting and sitting on a comfortable chair, as a control; (2) aerobic cycling exercise (CE); and (3) sAGPA (Figure 2). The CE was performed on a bicycle ergometer (75XL III; Konami, Tokyo, Japan) at the same heart rate level as that reached during the sAGPA trial (target heart rate: 117 ± 17 beats/min), which was determined for each participant during a pre-test. The intensity (measured in watts) was automatically adjusted to ensure that the participant’s heart rate corresponded to the target heart rate throughout the trial. The pedaling rate was kept constant at 60 rpm for all volunteers. SAGPA was performed in an artificially prepared, mock gardening space in our laboratory and comprised five types of AGPA: using a hoe by swinging, shoveling, pushing dirt back and forth, furrowing, and planting an artificial flower. This sequence was repeated four times during the 40-min trial (Figure 2). During each trial, heart rate, oxygen uptake, carbon dioxide output, expired minute ventilation, and respiratory exchange ratio were monitored continuously using a heart rate monitor (Polar V800; Polar Electro Japan Co., Ltd., Tokyo, Japan). We used an automatic gas analyzer that uses the mixing chamber method (AR10; Arco System, Chiba, Japan), which was calibrated before each test according to the manufacturer’s instructions. Heart rate at both baseline and during the trials was averaged every minute. Expired gas data at baseline and during the trials were averaged every 2 and 4 min, respectively. Intensity (watts) during the CE was recorded every 5 min. Exercise intensity, heart rate, and gas data during each 40-min trial were averaged as representative data. Metabolic equivalents were calculated as the representative oxygen uptake value during the trial divided by that at baseline. Before and 5 min after each trial, we measured the physiological variables of blood values, arterial stiffness, stress, and cognitive function.

#### 2.2.4 Physiological outcome measurements

Pulse wave velocity (PWV), cardio-ankle vascular index (CAVI), blood pressure, heart rate, and the pegboard test were measured for 30 s, as described previously (Nishiwaki et al., 2014). Saliva amylase activity (Saliva Amylase Monitor; Nipro, Osaka, Japan) was used for the stress evaluation.

The Flanker task and Stroop test were performed using a selective reaction time testing device (T. K. K. M-16051 and 23-inch PC monitor; Takei Scientific Instruments) to evaluate executive function. As described in a previous study (Bergelt et al., 2020), the modified Flanker task consisted of five arrowheads displayed on the monitor screen. The participant was asked to respond to the direction of the center target arrow by pressing the left button if the arrow pointed left and the right button if the arrow pointed right. The flanking arrows could point in the same direction (congruent condition) or in the direction opposite to the center arrow (incongruent condition). Each test session comprised 40 trials (20 congruent and 20 incongruent trials) presented in random order. Each trial was separated by an interstimulus interval, during which a fixed blank screen was presented for 2 s. The stimuli were presented on the screen for 2.5 s. The mean reaction time and accuracy rate were recorded for each condition, and the reaction time difference between the incongruent and congruent conditions was used as an index of Flanker interference.

As described previously (Kuwamizu et al., 2021), in the color-word Stroop test, two rows of letters were presented on the screen, and the participant was asked to indicate whether the color of the letters in the top row corresponded to the color name shown in the bottom row. Participants pressed either the left or right button to indicate a “yes” or “no” response, respectively. Each test session comprised 30 trials (10 neutral, 10 congruent, and 10 incongruent trials) presented in random order. Each trial was separated by an interstimulus interval, during which a fixed cross was presented on the screen for 9–13 s to avoid anticipation of the timing of the following trial. The stimuli were presented on the screen for 2 s. All words were written in Japanese. Mean reaction time and accuracy rate were recorded for each condition, and the reaction time difference between the incongruent and congruent conditions was used as an index of Stroop interference.

Venous blood samples were obtained from the antecubital vein. For serum analysis [cholesterol, triglycerides, free fatty acids, insulin, high-sensitivity C-reactive protein, the stable end-product of nitric oxide (NOx), and endothelin-1], blood was collected in serum separator tubes, allowed to clot at room temperature for 30 min, and then centrifuged at 1500 × *g* for 10 min at 25 °C. For plasma analysis, blood was collected in separate tubes. Samples for fibrinolytic markers [plasmin-α2 plasmin inhibitor complexes (PIC), total plasminogen activator inhibitor-1 (t-PAI-1), and D-dimer] were collected in sodium citrate tubes, whereas samples for catecholamines, adrenocorticotropic hormone, brain-derived neurotrophic factor (BDNF), and asymmetric dimethylarginine (ADMA) were collected in ethylenediaminetetraacetic acid-Na-coated tubes. Glucose was measured from samples collected in fluoride oxalate tubes. These plasma tubes were immediately placed on ice and centrifuged at 1500 × *g* for 10 min at 25 °C. All resulting serum and plasma samples were stored at −80 °C until use. The BDNF concentrations in plasma were measured using an enzyme-linked immunosorbent assay (ELISA) kit (R&D Systems, Minneapolis, MN, United States). Concentrations of circulating nitrite/nitrate (measured as NOx) and endothelin-1 in serum were also measured using an ELISA kit (R&D Systems). Concentrations of ADMA in plasma were measured using an ELISA kit (Immundiagnostik AG, Bensheim, Germany). We then calculated the ratio of NOx to ADMA. All measurements other than those assessed using ELISA kits were conducted by a commercial laboratory (LSI Medience Corporation, Tokyo, Japan), adhering to standardized clinical protocols. Because data for one measurement were missing, and the participant declined blood collection, BDNF was analyzed in samples from 10 participants only.

### 2.3 Hospital-based pair-matched data cross-sectional study

#### 2.3.1 Objectives

We aimed to clarify whether consistent AGPA affects neurovascular aging according to MRI, stroke prevalence, and cognitive function.

#### 2.3.2 Participants

**Figure 3:**
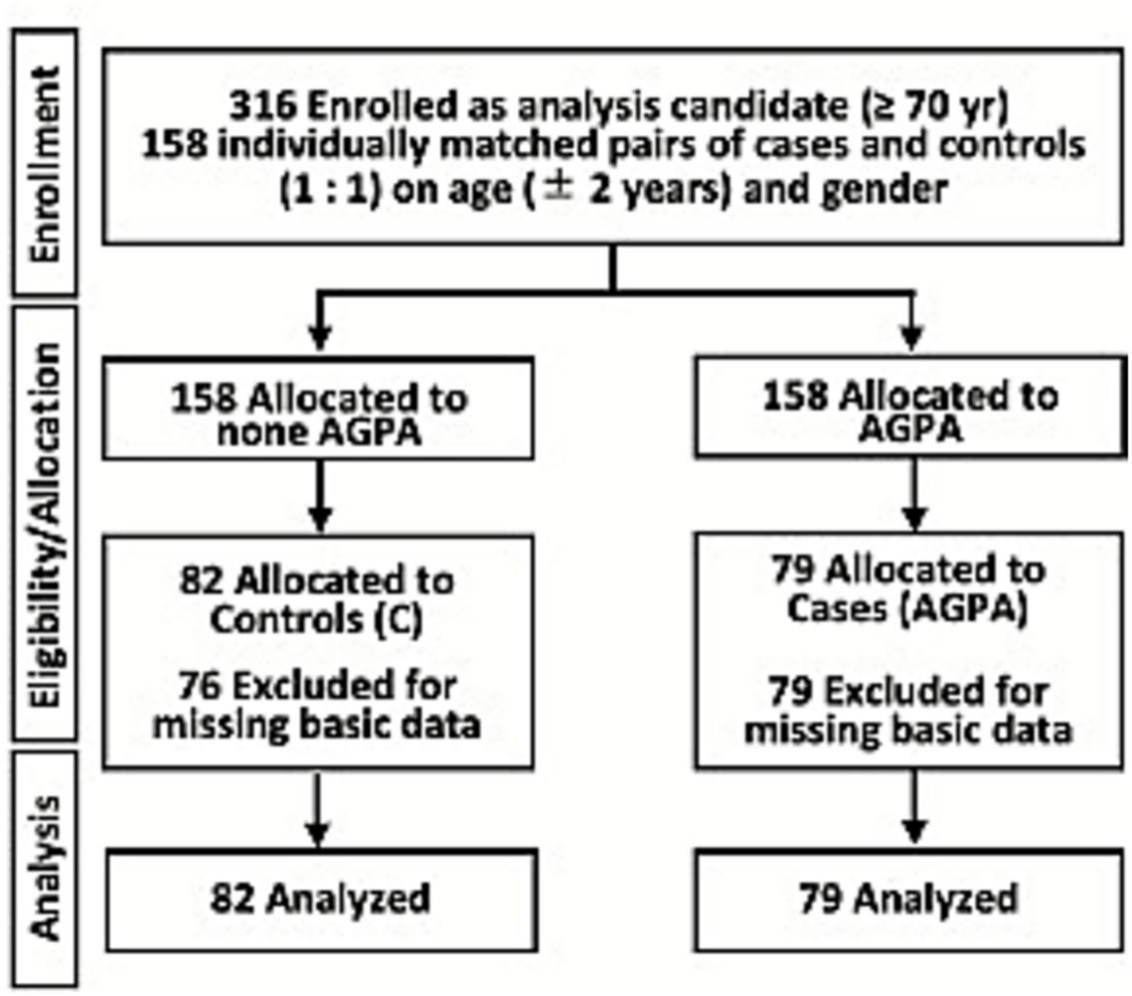
Flow diagram of the hospital-based pair-matched cross-sectional study. To explore the preventive effects of agricultural or gardening physical activity (AGPA) on neurovascular aging, we used cross-sectional data. C, controls; AGPA, agricultural or gardening physical activity.

Participants were identified from the electronic health records of Miyazaki Hospital between January 2018 and December 2018 who had complete brain MRI and cognitive function assessments available (i.e., no missing data), and who provided consent to participate in the study (Figure 3). The data were obtained after individuals were identified from the hospital data. The MRI data were analyzed retrospectively from records collected during routine clinical care. For allocation of participants to the AGPA and non-AGPA groups, we first identified the AGPA group and randomly recruited people of the same sex and age who visited the hospital on the same day (according to electronic health records) as the non-AGPA group. If there were no suitable participants for the non-AGPA group that visited on the same day, we expanded the search to the days before or after. To identify individuals to be allocated to the two groups, we checked records for the response to the question, “Do you regularly do farm work or gardening?” All participants were regular outpatients at the hospital. After group allocation, participants were asked again to provide consent for participation in the study and respond to additional questions. The data were accessed and analyzed for research purposes on March 1, 2019. We identified 161 Japanese adults ≥ 70 years of age, who were all Asian and met the inclusion criteria. Of the 161 adults, 79 were allocated to the AGPA group, and 82 age- (± 2 years) and sex-matched controls were allocated to the non-AGPA control group. Individuals in the AGPA group had engaged in AGPA 3.6 ± 2.4 times/week for 2.7 ± 1.6 h/day.

#### 2.3.3 Main outcome measurements

MRI data were acquired using standardized methods on a 1.5 T MAGNETOM Symphony Syngo or MAGNETOM Avanto scanner (Siemens Healthcare, Erlangen, Germany) to identify periventricular hyperintensities (PVHs) and deep and subcortical WMHs (DSWMHs), which were graded on a 5-point scale ranging from 0 to 4 (Manolio et al., 1994). PVH and DSWMH evaluations were performed by three independent, experienced neurosurgeons from different institutions who were blinded to patients’ clinical data. Cognitive function was assessed using the Hasegawa Dementia Scale-Revised (HDS-R), which is widely used in Japan and correlates strongly with the Mini-Mental State Examination, owing to several shared question items (Black et al., 2019). The HDS-R comprises nine questions that evaluate the perception of age, time, and place; the ability to repeat and recall words and objects, subtract serially, and count backward; category fluency; and confrontation naming ability. The different questions are scored a varied number of points, with a maximum score of 30 points (Chen and Juang, 2014). The cutoff value for the HDS-R is generally 20/21 points; a score below 20 is considered suspicious for dementia. In patients with stroke, the type of cerebral stroke (i.e., ischemic or hemorrhagic) was recorded.

#### 2.3.4 Covariates

We recorded age, sex, height, weight, BMI, sitting blood pressure (HBP-1300; Omron, Kyoto, Japan), circulating hemoglobin concentration (XT-2000i automated hematology analyzer; Sysmex, Kobe, Japan), and medications as clinical metrics. Exercise and physical activity status, smoking and drinking status, marital status, and educational level were assessed using a questionnaire, as described previously (Ogawa et al., 2020).

### 2.4 Statistical analysis

The results are presented as means ± standard deviations. For the experimental study, changes in parameters were evaluated using a two-way repeated-measures ANOVA, followed by Bonferroni correction for the *post hoc* multiple comparisons and simple main effects tests. Percentage change for all measured parameters described in the methods for the experimental study (i.e., baseline vs. post-experiment) was assessed using a one-way repeated-measures ANOVA (parametric) and the Friedman test (non-parametric), followed by the Bonferroni method. Statistical trends were analyzed using the Jonckheere–Terpstra test. For the cross-sectional study, continuous data were analyzed using independent t-tests and analysis of covariance (ANCOVA), including covariates. Differences in non-parametric variables were analyzed using the Mann–Whitney U test. Logistic regression was used to estimate odds ratios and 95% confidence intervals for the prevalence of stroke and dementia, adjusted for covariates in the multivariable model. These covariates were selected because previous studies suggested associations with stroke prevalence (Manolio et al., 1994; Wardlaw et al., 2017). Sensitivity analyses were also performed to verify the robustness of the results (similar results were obtained by performing comparisons of other pair-matched groups). For all statistical analyses, we used pairwise deletion methods (available-case analysis) if missing data were included in the main outcomes or covariates. Statistical analyses were conducted using SPSS version 25.0 J (IBM SPSS Japan, Tokyo, Japan), Excel Statistics 2015 (Social Survey Research Information, Tokyo, Japan), and Prism version 9.2.0 (GraphPad Software, San Diego, CA, United States). As previously described (Nishiwaki et al., 2014; Nishiwaki et al., 2018; Nishiwaki et al., 2019; Ogawa et al., 2020), the coefficients of variation, as a measure of reproducibility, for all of our measured parameters on two separate days were < 10%. Effect size and statistical power (1 − *β*) were calculated using G*Power 3. *p* < 0.05 was considered statistically significant.

## 3 Results

### 3.1 Experimental study of healthy adults using sAGPA

#### 3.1.1 Physiological and exercise parameters

Average heart rate was significantly higher in both the CE (116 ± 17 beats/min) and sAGPA (114 ± 19 beats/min) trials than in the control trial (64 ± 9 beats/min; *p* < 0.001; Table 1). However, there was no significant difference in average heart rate between the CE and sAGPA trials. There were also no significant differences in metabolic equivalents between the CE and sAGPA trials (Table 1).

**Table 1:** Physiological parameters at baseline and during the trial.

Parameter	C	CE	sAGPA	P (ANOVA)
HR at baseline, beats/min	68 ± 11	74 ± 13	76 ± 15	0.109
HR during trial, beats/min	64 ± 9	116 ± 17**	114 ± 19**	< 0.001
VO_2_ at baseline, mL/min	291 ± 96	304 ± 74	315 ± 107	0.800
VO_2_ during the trial, mL/min	236 ± 29	1,149 ± 195**	849 ± 192**††	< 0.001
VCO_2_ at baseline, mL/min	242 ± 94	256 ± 63	265 ± 68	0.682
VCO_2_ during the trial, mL/min	193 ± 24	1,046 ± 178**	786 ± 197*††	< 0.001
VE at baseline, L/min	10.25 ± 4.14	9.58 ± 2.66	9.68 ± 2.28	0.782
VE during the trial, L/min	7.11 ± 0.89	28.10 ± 5.94**	23.78 ± 6.84**	< 0.001
RER at baseline, unit	0.84 ± 0.27	0.86 ± 0.17	0.88 ± 0.20	0.600
RER during the trial, unit	0.82 ± 0.05	0.91 ± 0.04**	0.93 ± 0.05**	< 0.001
Cycling intensity, Watts	–	70 ± 20	–	
METs, unit	0.9 ± 0.3	3.9 ± 0.8**	3.0 ± 1.4**	< 0.001

Data are expressed as means ± standard deviations. **p < 0.01 vs. the C trial, ††*p* < 0.01 vs. the CE trial. C, control trial; CE, cycling exercise trial; sAGPA, simulated agricultural or gardening physical activity trial; ANOVA, analysis of variance; HR, heart rate; VO_2_, oxygen uptake; VCO_2_, carbon dioxide output; VE, expired minute ventilation; RER, respiratory exchange ratio; METs, metabolic equivalents.

#### 3.1.2 Arterial stiffness, stress, and cognitive function

**Figure 4:**
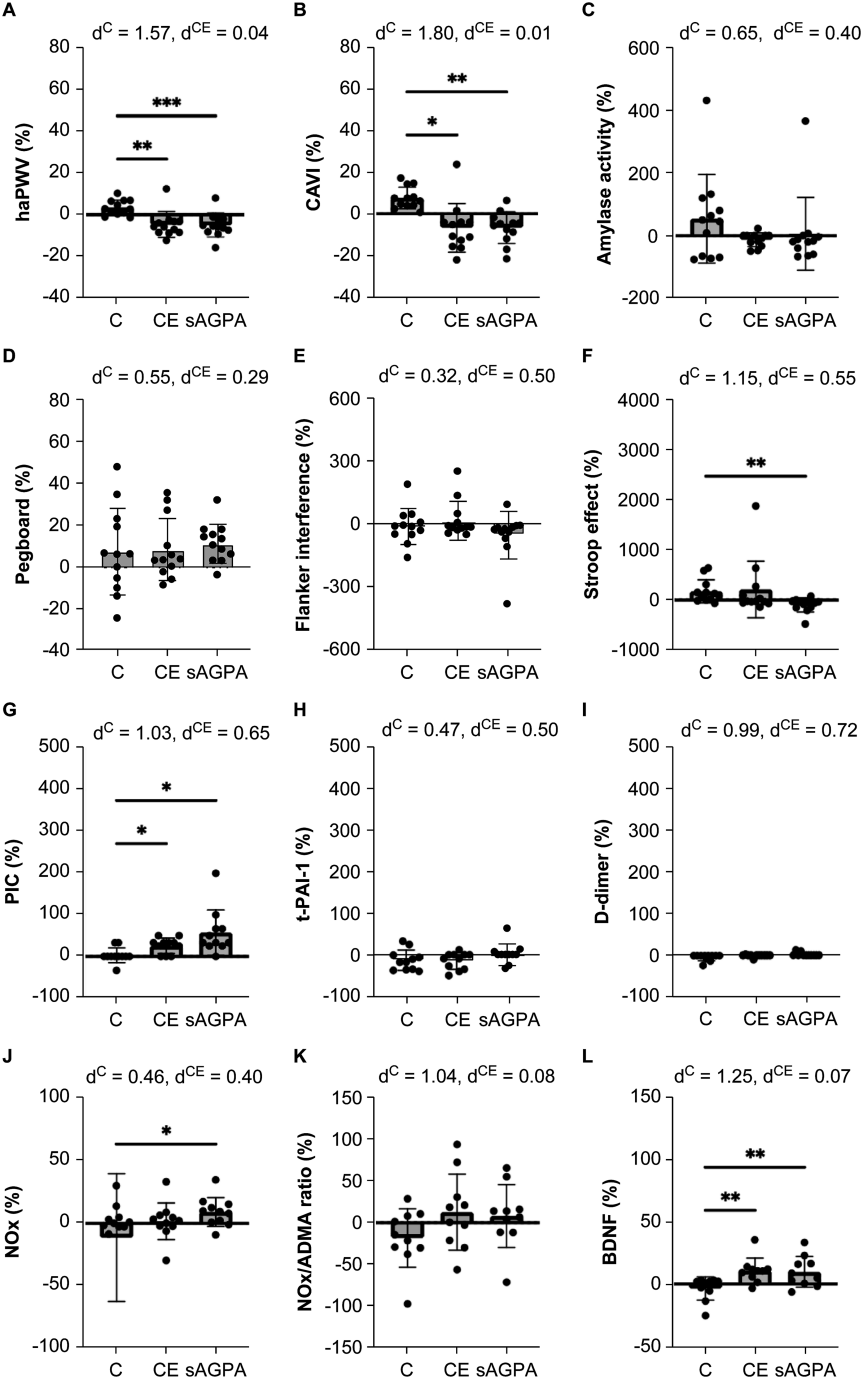
Arterial stiffness **(A,B)**, cognition-related parameters **(C–F)**, and circulation-related parameters **(G–L)** before and after the single-session exercise protocols. Both the single-session cycling exercise (CE) and simulated agricultural or gardening physical activity (sAGPA) reduced arterial stiffness and increased plasmin-*α*2-plasmin inhibitor complex (PIC) and brain-derived neurotrophic factor (BDNF) levels. Furthermore, alterations in the Stroop effect and NOx concentration were observed only in the sAGPA group. Blood samples were analyzed for 10 of 11 participants owing to missing data and refusal to provide a blood sample. Data are expressed as means ± standard deviations. haPWV, heart-ankle pulse wave velocity; CAVI, cardio-ankle vascular index; PIC, plasmin-α2 plasmin inhibitor complexes; t-PAI-1, total plasminogen activator inhibitor-1; NOx, stable end-product (nitrite/nitrate) of nitric oxide; ADMA, asymmetric dimethylarginine; BDNF, brain-derived neurotrophic factor; C, control group; CE, cycling exercise group; P^D^, *p*-value for statistical differences; P^T^, p-value for statistical trends; d^c^, effect sizes between the control and sAGPA trials; d^CE^, effect sizes between the CE and sAGPA trials; *p < 0.05 vs. C trial; **p < 0.01 vs. C trial.

The mean heart-ankle PWV and CAVI values decreased significantly after the CE (pre: 609 ± 39 cm/s, post: 579 ± 28 cm/s, *p* < 0.05; and pre: 614 ± 37 units, post: 584 ± 56 units, *p* < 0.05; respectively) and sAGPA (pre: 6.1 ± 0.6 cm/s, post: 5.6 ± 0.5 cm/s, *p* < 0.05; and pre: 6.1 ± 0.5 units, post: 5.7 ± 0.7 units, *p* < 0.05; respectively) trials, but not after the control trial (Table 2). Furthermore, reductions were significantly higher in the CE and sAGPA trials than in the control trial (*p* < 0.01 and *p* < 0.001, respectively; Figure 4). We also observed a significant increase in pegboard test scores (pre: 29 ± 4 points, post: 33 ± 4 points, *p* < 0.05), respectively, following the sAGPA trial (Table 2). The accuracy rate of the Flanker task did not change; however, mean reaction times of the congruent and incongruent conditions shortened significantly following the sAGPA trial (pre: 460 ± 182 ms, post: 429 ± 15α-amylase activity and decrease and ms, *p* < 0.05; and pre: 505 ± 180 ms, post: 467 ± 157 ms, *p* < 0.05; respectively; Table 2). Accuracy and reaction time of the congruent condition of the Stroop test also showed no significant changes following the sAGPA trial; however, mean reaction times of the incongruent condition shortened (pre: 920 ± 201 ms, post: 817 ± 238 ms, *p* < 0.05), and the Stroop effect decreased significantly (pre: 120 ± 101 ms, post: 41 ± 127 ms, *p* < 0.05; Table 2). Additionally, the reduction in the Stroop effect following the sAGPA trial was significantly greater than that following the control trial (*p* < 0.01; Figure 4).

**Table 2:** Physiological and psychological parameters before and after each acute experiment trial.

Parameters	C		CE		sAGPA		Int. P
	Pre	Post	Pre	Post	Pre	Post	
HR, beats/min	62 ± 8	58 ± 8	61 ± 11	70 ± 16*	63 ± 15	66 ± 15	< 0.001
SBP, mmHg	119 ± 4	118 ± 5	118 ± 6	120 ± 5	121 ± 7	119 ± 7	0.136
DBP, mmHg	71 ± 6	71 ± 6	69 ± 4	68 ± 7	68 ± 8	66 ± 6	0.957
haPWV, cm/s	614 ± 28	635 ± 33*	609 ± 39	579 ± 28*	614 ± 37	584 ± 56*	< 0.001
CAVI, unit	6.1 ± 0.4	6.6 ± 0.5*	6.1 ± 0.6	5.6 ± 0.5*	6.1 ± 0.5	5.7 ± 0.7*	< 0.001
Stress, kU/L	13.5 ± 9.6	13.8 ± 11.0	15.8 ± 16.6	14.2 ± 17.2	20.0 ± 16.8	17.0 ± 16.1	0.623
Pegboard, score	31 ± 5	32 ± 3	31 ± 4	34 ± 4	29 ± 4	33 ± 4*	0.638
F. accuracy, %	98.7 ± 2.0	98.8 ± 2.0	98.5 ± 2.9	98.3 ± 1.7	97.0 ± 5.0	98.8 ± 1.7	0.293
F. congruent, ms	426 ± 142	404 ± 97	403 ± 92	373 ± 61	460 ± 182	429 ± 15*	0.905
F. incongruent, ms	465 ± 132	448 ± 98	461 ± 116	424 ± 69*	505 ± 180	467 ± 157*	0.592
F. interference, ms	39 ± 63	45 ± 26	58 ± 34	51 ± 21	45 ± 27	38 ± 24	0.701
S. accuracy, %	96.5 ± 4.2	97.2 ± 2.4	96.9 ± 3.6	97.5 ± 2.9	96.3 ± 5.8	98.6 ± 2.2	0.525
S. congruent, ms	737 ± 166	690 ± 155	669 ±156	652 ± 140	800 ± 180	777 ± 246	0.693
S. incongruent, ms	774 ± 192	769 ± 180	735 ± 184	720 ± 175	920 ± 201	817 ± 238*	0.023
S. effects, ms	38 ± 52	80 ± 67*	66 ± 81	68 ± 59	120 ± 101	41 ± 127*	0.004
HDL, mg/dL	51 ± 9	52 ± 10	51 ± 11	54 ± 11*	48 ± 10	52 ± 10*	< 0.001
LDL, mg/dL	98 ± 24	100 ± 25	98 ± 24	104 ± 27*	90 ± 20	98 ± 24*	0.009
TG, mg/dL	83 ± 33	68 ± 22	103 ± 54	97 ± 59	94 ± 39	92 ± 43	0.432
FFA, eEq/L	0.33 ± 0.20	0.36 ± 0.26	0.35 ± 0.20	0.64 ± 0.15*	0.39 ± 0.40	0.56 ± 0.42*	< 0.001
Glucose, mg/dL	105 ± 25	98 ± 7	97 ± 10	95 ± 6	101 ± 16	93 ± 10	0.588
Insulin, μU/mL	11.4 ± 17.0	7.3 ± 7.6	7.9 ± 4.4	5.5 ± 2.8	9.8 ± 11.4	4.7 ± 3.4	0.809
Noradrena., ng/mL	0.24 ± 0.07	0.26 ± 0.08	0.31 ± 0.13	0.44 ± 0.17*	0.16 ± 0.08	0.31 ± 0.16*	0.002
Adrena., ng/mL	0.05 ± 0.02	0.04 ± 0.02	0.06 ± 0.03	0.08 ± 0.03	0.04 ± 0.02	0.06 ± 0.04*	0.057
hsCRP, mg/dL	0.086 ± 0.143	0.085 ± 0.143	0.045 ± 0.065	0.053 ± 0.076*	0.037 ± 0.054	0.044 ± 0.066	0.151
Cortisol, μg/dL	9.2 ± 3.7	7.7 ± 3.5	8.6 ± 1.6	8.2 ± 2.9	8.8 ± 3.3	8.3 ± 4.0	0.702
ACTH, pg./mL	24.2 ± 12.0	19.9 ± 9.8	20.6 ± 7.1	22.2 ± 9.2	21.4 ± 6.6	24.7 ± 15.0	0.185
PIC, μg/mL	0.33 ± 0.06	0.34 ± 0.08	0.39 ± 0.09	0.49 ± 0.14*	0.35 ± 0.09	0.55 ± 0.18*	0.002
t-PAI-1, ng/mL	17.0 ± 7.3	14.1 ± 4.2	15.3 ± 5.5	12.7 ± 5.6	18.9 ± 14.9	17.7 ± 10.3	0.701
D-dimer, μg/mL	0.40 ± 0.19	0.37 ± 0.16*	0.33 ± 0.06	0.33 ± 0.06	0.31 ± 0.04	0.32 ± 0.05	0.040
NOx, μmol/L	10.0 ± 4.1	9.4 ± 4.9	12.9 ± 6.3	13.2 ± 7.0	10.5 ± 5.3	11.7 ± 4.5*	0.279
ADMA, μmol/L	0.23 ± 0.04	0.27 ± 0.06*	0.25 ±0.05	0.25 ± 0.06	0.26 ± 0.06	0.27 ± 0.03	0.407
NOx/ADMA ratio	44.7 ± 19.5	34.4 ± 17.6*	56.5 ± 34.3	56.8 ± 33.6	45.0 ± 28.8	42.8 ± 14.8	0.366
Endothelin, pg./mL	2.7 ± 2.7	2.8 ± 1.7	2.6 ± 2.2	3.2 ± 2.0	2.8 ± 1.9	2.9 ± 1.6	0.856
BDNF, pg./mL	1,315 ± 773	1,304 ± 1,019	797 ± 503	1,351 ± 488	1,382 ± 970	2,750 ± 3280*	0.151

Blood samples were analyzed for 10 of 11 participants because of missing data and refusal to provide a blood sample in one patient. Data are expressed as means ± standard deviations. *p < 0.05 for the interaction between the Pre and Post values; one-way repeated-measures analysis of variance (parametric) or the Friedman test (non-parametric), followed by the Bonferroni method. C, control trial; CE, cycling exercise trial; sAGPA, simulated agricultural or gardening physical activity trial; HR, heart rate; SBP, systolic blood pressure; DBP, diastolic blood pressure; haPWV, heart-ankle pulse wave velocity; CAVI, cardio-ankle vascular index; Stress, salivary α-amylase activity; F, Flanker test; S, Stroop test; HDL, high-density lipoprotein; LDL, low-density lipoprotein; TG, triglyceride; FFA, free fatty acid; Noradren, noradrenaline; Adren, adrenaline; hsCRP, high-sensitivity C-reactive protein; ACTH, adrenocorticotropic hormone; PIC, plasmin-α2-plasmin inhibitor complexes; t-PAI-1, total plasminogen activator inhibitor-1; NOx, stable end-product of nitric oxide (nitrite/nitrate); Endothelin, endothelin-1; ADMA, asymmetric dimethylarginine; BDNF, brain-derived neurotrophic factor; Int. P, interaction *p*-value.

#### 3.1.3 Circulating biomarkers

Mean high-density lipoprotein, low-density lipoprotein, free fatty acid, and noradrenaline concentrations increased significantly following both the CE (pre: 51 ± 11 mg/dL, post: 54 ± 11 mg/dL, *p* < 0.05; pre: 98 ± 24 mg/dL, post: 104 ± 27 mg/dL, *p* < 0.05; pre: 0.35 ± 0.20 eEq/L, post: 0.64 ± 0.15 eEq/L, *p* < 0.05; and pre: 0.31 ± 0.13 ng/mL, post: 0.44 ± 0.17 ng/mL, *p* < 0.05; respectively) and sAGPA (pre: 48 ± 10 mg/dL, post: 52 ± 10 mg/dL, *p* < 0.05; pre: 90 ± 20 mg/dL, post: 98 ± 24 mg/dL, *p* < 0.05; pre: 0.39 ± 0.40 eEq/L, post: 0.56 ± 0.42 eEq/L, *p* < 0.05; and pre: 0.16 ± 0.08 ng/mL, post: 0.31 ± 0.16 ng/mL, *p* < 0.05; respectively) trials (Table 2). Although adrenaline concentrations tended to increase following the CE trial, a significant increase in adrenaline concentration was found only following the sAGPA trial (pre: 0.04 ± 0.02 ng/mL, post: 0.06 ± 0.04 ng/mL, *p* < 0.05; Table 2). Interestingly, mean PIC increased significantly after both the CE and sAGPA trials (pre: 0.39 ± 0.09 μg/mL, post: 0.49 ± 0.14 μg/mL, *p* < 0.05; and pre: 0.35 ± 0.09 μg/mL, post: 0.55 ± 0.18 μg/mL, *p* < 0.05; respectively); moreover, there was a significantly greater increase in PIC following the sAGPA trial than following the control trial (*p* < 0.05; Table 2 and Figure 4). However, *t*-PAI-1 and D-dimer levels showed no significant changes after the CE or sAGPA trials. NOx concentrations increased significantly exclusively after the sAGPA trial (pre: 10.5 ± 5.3 μmol/L, post: 11.7 ± 4.5 μmol/L, p < 0.05), and this increase was significantly greater in the sAGPA trial than in the control trial (*p* < 0.05; Table 2 and Figure 4). However, no significant changes in endothelin-1 or ADMA were observed for any of the trial types. BDNF concentrations increased significantly only following the sAGPA trial (pre: 1382 ± 970 pg./mL, post: 2750 ± 3,280 pg./mL, *p* < 0.05), and this increase was significantly greater following the sAGPA and CE trials than following the control trial (both *p* < 0.01; Table 2; Figure 4).

### 3.2 Hospital-based pair-matched data cross-sectional study

**Figure 5:**
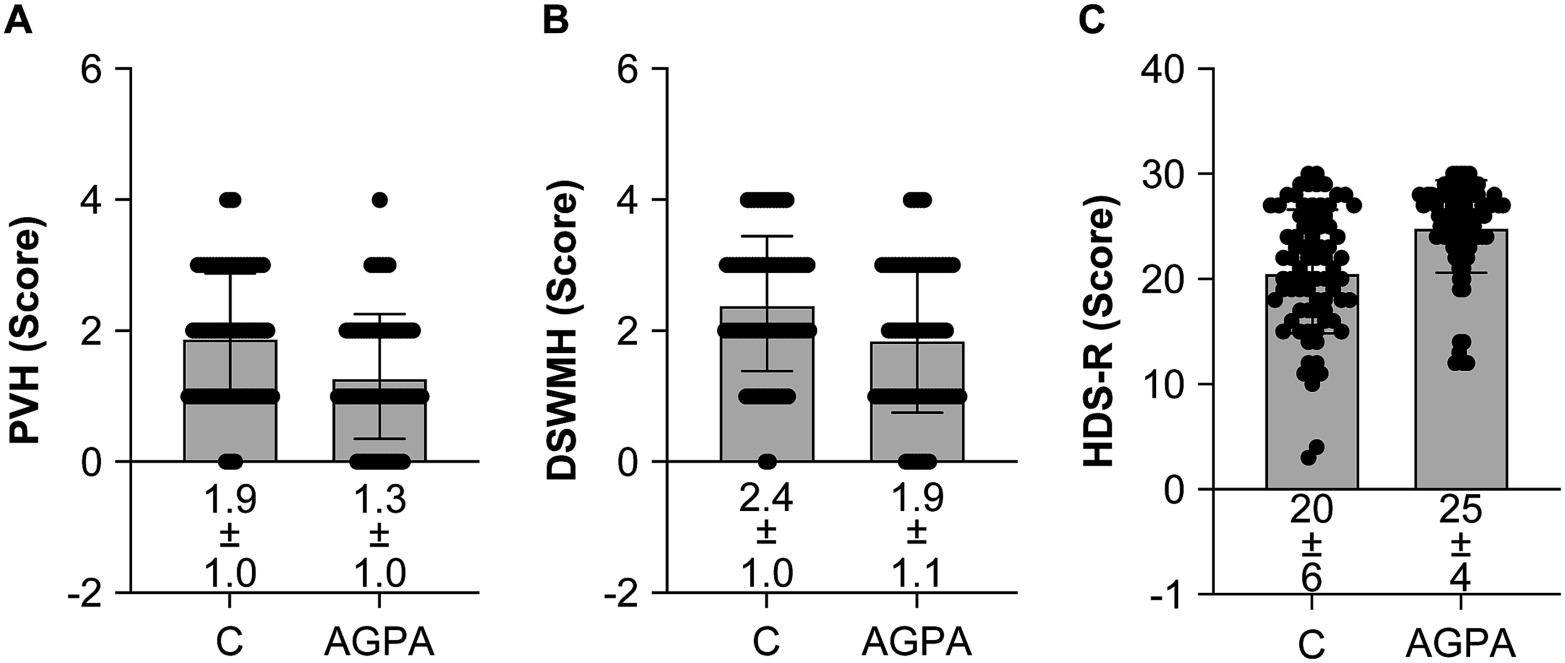
White matter hyperintensities and cognitive function of participants in the hospital-based pair-matched cross-sectional study. **(A)** Periventricular hyperintensities (PVH) were significantly lower in the agricultural or gardening physical activity (AGPA) group than in the control **(C)** group [*t*-test *p* < 0.001, effect size (ES) (d) = 0.60, analysis of covariance (ANCOVA) *p* = 0.002, ES (ƞ_p_^2^) = 0.070]. **(B)** Deep and subcortical white matter hyperintensities (DSWMH) were significantly lower in the AGPA group than in the C group [*t*-test *p* = 0.002, ES (d) = 0.476, ANCOVA *p* = 0.02, ES (ƞ_p_^2^) = 0.038]. **(C)** Hasegawa Dementia Scale-Revised (HSD-R) scores were significantly higher in the AGPA group than in the C group [*t*-test *p* < 0.001, ES (d) = 0.828, ANCOVA *p* < 0.001, ES (ƞ_p_^2^) = 0.121]. The ANCOVA included age, sex, body mass index, mean blood pressure, antiplatelet agents, anticoagulant agents, hypertension, diabetes mellitus, hyperlipidemia, dementia, osteoporosis, sleep medication, exercise and physical activity habits, current smoker status, alcohol consumption, marital status, and educational attainment as covariates. For the ANCOVA, *n* = 82 and 74 for the C and APGA groups, respectively. For the *t*-test, *n* = 82 and 79 for the C and APGA groups, respectively. Data are expressed as means ± standard deviations.

No significant differences in age, height, weight, BMI, blood pressure, physical activity, smoking status, or alcohol consumption were observed between the AGPA group and controls (Table 3). Furthermore, hemoglobin levels (control: 13.3 ± 1.3 g/dL, AGPA: 13.8 ± 1.2 g/dL; *p* = 0.021) and exercise habits (control: 30.5%, AGPA: 53.2%, *p* = 0.005) were significantly poorer in the control group than in the AGPA group (Table 3). Moreover, the control group was taking more antiplatelet agents (control: 74.4%, AGPA: 57.0%, *p* = 0.020), anti-hypertensive medications (control: 58.5%, AGPA: 32.9%, *p* = 0.001), and sleep medications (control: 25.6%, AGPA: 8.9%, *p* = 0.005) than the AGPA group (Table 3). The AGPA group had a significantly higher proportion of individuals who were married (AGPA: 79.5%, control: 63.4%) but had a significantly lower education level (<9 years, AGPA: 57.7%, control: 39.0%; ≥13 years, AGPA: 9.0%, control: 15.9%) than the control group (Table 3). Notably, PVH (AGPA: 1.3, control: 1.9) and DSWMH grades (AGPA: 1.9, control: 2.4) were significantly lower, and HDS-R scores (AGPA: 25, control: 20) were significantly higher in the AGPA group than in the control group, with and without adjusting for covariates (Figure 5). Crucially, total cerebral stroke prevalence was significantly higher in the control group than in the AGPA group (with and without adjusting for confounding factors), which was attributed to a tendency toward higher ischemic stroke prevalence in the control group (Table 4). The logistic regression analysis indicated that the full adjusted odds ratio was 4.88 (95% confidence interval: 1.67–14.28), and a lifestyle without habitual AGPA was significantly more strongly associated with a higher prevalence of total stroke (ischemic and hemorrhagic stroke) than a lifestyle with habitual AGPA (Table 4). The logistic regression analysis indicated that the full adjusted odds ratio was 5.08 (95% confidence interval: 2.02–12.80), and a lifestyle without habitual AGPA was significantly more strongly associated with a higher prevalence of dementia than a lifestyle with habitual AGPA (Table 4).

**Table 3:** Characteristics of the hospital-based pair-matched cross-sectional study participants.

Parameter	C group	AGPA group	*p*-value
Participants, *n* (men/women)	82 (47/35)	79 (54/25)	0.149
Age, years	78 ± 5	78 ± 5	0.480
Height, cm	155.5 ± 9.2	157.9 ± 7.5	0.064
Weight, kg	55.3 ± 11.1	56.7 ± 8.3	0.362
BMI, kg/m^2^	22.8 ± 3.3	22.7 ± 2.7	0.929
Systolic BP, mmHg	133 ± 18	135 ± 16	0.577
Diastolic BP, mmHg	73 ± 10	74 ± 10	0.494
Hemoglobin, g/dL	13.3 ± 1.3	13.8 ± 1.2	0.021
Medication status			
Antiplatelet agents, *n* (%)	61 (74.4)	45 (57.0)	0.020
Anticoagulant agents, *n* (%)	7 (8.5)	7 (8.9)	0.942
Hypertension, *n* (%)	48 (58.5)	26 (32.9)	0.001
Diabetes mellitus, *n* (%)	13 (15.9)	6 (7.6)	0.106
Hyperlipidemia, *n* (%)	29 (35.4)	21 (26.6)	0.230
Dementia, *n* (%)	24 (29.3)	24 (30.4)	0.878
Osteoporosis, *n* (%)	8 (9.8)	2 (2.5)	0.058
Sleep medication, *n* (%)	21 (25.6)	7 (8.9)	0.005
Exercise habits, *n* (%)	25 (30.5)	41 (53.2)	0.005
Physical activity habits, *n* (%)	46 (56.1)	53 (67.9)	0.124
Current smoker, *n* (%)	5 (6.1)	1 (1.3)	0.355
Alcohol consumption	*n* = 82	*n* = 78	0.240
Non-drinker, *n* (%)	65 (79.3)	55 (70.5)	–
Moderate drinker, *n* (%)	13 (15.9)	20 (25.6)	–
Heavy drinker, *n* (%)	4 (4.9)	3 (3.8)	–
Marital status	*n* = 82	*n* = 78	0.021
Married, *n* (%)	52 (63.4)	62 (79.5)	–
No data, *n* (%)	28 (34.1)	16 (20.5)	–
Single, *n* (%)	2 (2.4)	0 (0.0)	–
Education level	82	78	0.017
< 9 years, *n* (%)	32 (39.0)	45 (57.7)	–
10–12 years, *n* (%)	37 (45.1)	26 (33.3)	–
≥ 13 years, *n* (%)	13 (15.9)	7 (9.0)	–

Data are expressed as means ± standard deviations. *p*-values indicate the results of independent *t*-tests or Mann–Whitney U tests. BMI, body mass index; BP, blood pressure; C, pair-matched control group; AGPA, agriculture and gardening physical activity.

**Table 4:** Odds ratios for the prevalence of stroke and dementia by AGPA status in the hospital-based pair-matched cross-sectional study.

	*n*	Cases	Model 1	Model 2	Model 3
Ischemic stroke					
AGPA	79	27	1.00 (Reference)	1.00 (Reference)	1.00 (Reference)
Control	82	50	3.23 (1.68–6.24)	3.23 (1.67–6.23)	2.38 (0.74–7.69)
Hemorrhagic stroke					
AGPA	79	5	1.00 (Reference)	1.00 (Reference)	1.00 (Reference)
Control	82	10	2.29 (0.72–7.25)	2.39 (0.74–7.74)	2.10 (0.39–11.34)
Total stroke					
AGPA	79	28	1.00 (Reference)	1.00 (Reference)	1.00 (Reference)
Control	82	57	4.68 (2.35–9.30)	4.78 (2.39–9.55)	4.88 (1.67–14.28)
Dementia					
AGPA	79	11	1.00 (Reference)	1.00 (Reference)	1.00 (Reference)
Control	82	40	6.35 (2.90–13.93)	6.33 (2.88–13.92)	5.08 (2.02–12.80)

Data are expressed as odds ratios (95% confidence intervals). Because of missing covariate data, the number of analyzed participants (n) for AGPA/Control in Model 3 was 74/82. The analyzed cases of AGPA/Control were 25/50 in ischemic stroke, 5/10 in hemorrhagic stroke, 26/57 in total stroke, and 11/40 in dementia. Model 1: Adjusted for age (years) and sex (men, women). Model 2: Adjusted for Model 1 plus body mass index and mean blood pressure. Model 3: Adjusted for Model 2 plus antiplatelet agents (yes, no), anticoagulant agents (yes, no), hypertension (yes, no), diabetes mellitus (yes, no), hyperlipidemia (yes, no), dementia (yes, no), osteoporosis (yes, no), sleep medication (yes, no), exercise habits (yes, no), physical activity habits (yes, no), current smoker status (yes, no), alcohol consumption (non-drinker, moderate drinker, heavy drinker), marital status (married, no data, single), and educational attainment (< 9 years, 10–12 years, ≥ 13 years). AGPA, agricultural or gardening physical activity.

## 4 Discussion

We found that both short-term CE and sAGPA induced a reduction in arterial stiffness and an increase in PIC and BDNF levels. Furthermore, favorable alterations in the Stroop effect and NOx levels were observed only following the sAGPA trial. For the second study, in addition to the higher cognitive function of the AGPA group, MRI data indicated that cerebral WMHs and stroke prevalence were lower in the AGPA group than in the control group. To the best of our knowledge, this is the first demonstration that the incidence of aging-associated brain diseases, such as stroke and dementia, can be lowered (via arterial stiffness reduction) by engaging in regular AGPA.

To detect specific responses to AGPA, we first conducted a short-term sAGPA intervention study. Because heart rate and metabolic equivalents did not differ between the CE and sAGPA trials, the intensity of activity was likely similar, despite the different modes. Consistent with previous studies on exercise, we observed a greater reduction in arterial stiffness following both activity trials than following the control trial. Greater PWV is generally associated with a poorer WMH grade, and effective management of increased PWV helps prevent the progression of WMHs (Kuo et al., 2010). Moreover, greater increases in PIC and BDNF levels, as indices of fibrinolytic activity/neural plasticity and central neuron survival, respectively, were found after both the CE and sAGPA trials than after the control trial. Low levels of BDNF are also related to a higher risk of ischemic and hemorrhagic stroke and poor recovery (Lasek-Bal et al., 2015; Stanne et al., 2016; Pedard et al., 2018; Chaturvedi et al., 2020). These alterations are likely attributed to the effects of physical activity in general, rather than AGPA specifically.

We also observed a significantly greater increase in PIC levels following the sAGPA trial (57.6% ± 54.3%) than after the CE trial (25.8% ± 18.4%), despite no significant changes in t-PAI-1 or D-dimer levels. This suggests that sAGPA increased plasmin without thrombus formation or lysis in the fibrinolytic system; thus, AGPA may induce hyperfibrinolysis and prevent future thrombus formation. Higher t-PAI-1 levels are associated with an increased risk of first-ever stroke, and disturbances in fibrinolysis precede cerebrovascular events (Johansson et al., 2000). Thus, the hyperfibrinolysis observed after sAGPA may help prevent issues related to neurovascular aging, such as low-grade WMHs and stroke. Notably, we observed greater alterations in NOx levels and the Stroop effect, as important indices of vasoactive substances and cognitive executive function, respectively, and a tendency toward a greater amylase activity reduction and pegboard score increase, as indices of stress and cognitive function-related dexterity, respectively, following the sAGPA trial than following the control trial. Therefore, these changes in hyperfibrinolysis, NOx levels, cognitive function, and stress status may be attributed to specific elements of AGPA. Taken together, AGPA may induce a combination of general physical activity-induced effects and specific AGPA-related effects; thus, AGPA may offer similar or even greater benefits than continuous CE.

Aerobic exercise, as an element of physical activity, increases blood flow and shear stress, and these repetitive alterations trigger vasodilator release and reduce arterial stiffness (Green et al., 2017; Tanaka, 2019). Blood flow and shear stress also induce an increase in t-PA, but not PAI-1, in the endothelium (Diamond et al., 1989). The change in balance between t-PA and PAI-1 subsequently increases plasmin, thereby eliciting hyperfibrinolysis, as reflected by an increase in PIC level. Previous studies have suggested that this hyperfibrinolysis induces the activation of BDNF, which improves neurological symptoms (Liu and Nusslock, 2018). Furthermore, BDNF has been identified as a contraction-induced myokine, and several studies have shown increases in circulating BDNF following short-term exercise or hypoxia (Delezie et al., 2019). Therefore, physical activity-induced muscle contraction or hypoxia can increase circulating BDNF levels. Additionally, exercise increases the level of BDNF in various brain regions and peripheral circulating insulin-like growth factor 1 (IGF-1), and this signaling manifests as an exercise-induced increase in hippocampal plasticity (Cotman et al., 2007). Therefore, one possibility is that increased BDNF in the brain and peripheral IGF-1 contribute to higher circulating levels of BDNF. Indeed, blockade of the BDNF receptor inhibits exercise-induced increases in cognition; moreover, synaptic protein levels and the administration of exogenous BDNF prevent aging-associated pathological changes in the nervous system, which highlights the critical role that BDNF plays in the coordination of physical activity-induced neural and cognitive changes (Casaletto et al., 2022).

The benefits can vary depending on the type of physical activity. In contrast to CE, which focuses on the lower limbs, AGPA is a systemic activity that uses both the upper and lower limbs. Greater participation of different muscles increases blood flow and shear stress, especially in the upper limbs; thus, NOx levels and hyperfibrinolysis in the endothelium may increase more readily following AGPA than CE. Indeed, a cohort study of 80,306 British adults revealed a significant reduction in cardiovascular disease mortality in those who engaged in swimming, racquet sports, and aerobics, whereas no significant associations were found for those who participated in cycling, running, or football (Oja et al., 2017). This finding suggests that the use of both the upper and lower limbs during exercise is a major factor in the prevention of death due to cardiovascular disease. Another study demonstrated that interval exercise increases the internal carotid artery shear rate more than the equivalent work volume of continuous exercise, and thus interval exercise may be more effective in improving cerebrovascular function, as shown by a decrease in the risk of cerebrovascular diseases (Ogoh et al., 2021). The similarity of AGPA (a largely non-continuous exercise) to interval and circuit exercises may contribute to the favorable effects of AGPA on cerebrovascular and cognitive functions.

Finally, hand and finger movements are a specific element of AGPA, used for activities such as planting seedlings or flowers, and such fine movements may improve dexterity and cognitive function. It has been demonstrated that dual-task exercise training increases cognitive functions more than exercise alone (Schwenk et al., 2010). Given that AGPA contains more elements of dual-task exercises than CE, we expected a greater improvement in the Flanker and Stroop test performance following AGPA. Furthermore, mental health benefits may be gained from exposure to soil, plants, and nature (daylight and breezes) during AGPA (Soga et al., 2016). Indeed, previous studies have reported that exposure to green space is associated with better mental health, self-reported health, and well-being (Astell-Burt and Feng, 2019; White et al., 2019). Therefore, the combined effects of general and specific elements of AGPA may help prevent or lower the risk of neurovascular aging. Previous observational data (Blair et al., 1992; Fleming et al., 1999; Stiernström et al., 2001; Blair et al., 2005; Matsumori et al., 2009; Waggoner et al., 2011; Armitage et al., 2012; Kawasaki, 2015; Levêque-Morlais et al., 2015; Brew et al., 2016; Lêng and Wang, 2016) have shown that engaging in agriculture or gardening activities enables better health and longevity; however, among the several factors in agriculture and gardening (e.g., physical activity, the natural environment, and diet), the physical activity element may be the sole contributor to such outcomes.

As illustrated by the Thomas Sydenham axiom that “man is as old as his arteries,” arterial health—more specifically, arterial stiffness/compliance—is widely considered a barometer of biological or physiological aging (Tanaka, 2019). Aging leads to an impairment of blood vessel function, and vascular dysfunction is a key characteristic of conditions associated with neurovascular aging, such as stroke and dementia (El Assar et al., 2012; Graham, 2017), which can have a detrimental impact on the quality of life of older adults. Therefore, maintaining healthy blood vessels is key to healthy aging.

In the second study, we conducted a single hospital-based pair-matched cross-sectional study in older adult patients to examine the effect of AGPA on the development of aging-associated brain diseases, such as stroke and dementia. Our findings suggested that AGPA has an anti-hypertensive effect, reduces cerebral WMHs, prevents stroke, and reduces cognitive decline. These effects of AGPA are consistent with a previous study in older Australian adults that showed that those who regularly garden have a 36% lower risk of dementia than their non-gardening counterparts (Simons et al., 2006). Reinforcing this finding, in a recent study of over 136,000 adults, gardening was associated with a lower likelihood of subjective cognitive decline—an effect that was partially mediated by increased physical activity and reduced psychological distress (Wang et al., 2024). Moreover, in our recent community-based pair-matched cross-sectional study of 30 individuals who performed AGPA and 30 age-, sex-, and objective activity-matched controls (Nishiwaki et al., 2025), arterial stiffness (PWV) was lower, and hand-finger dexterity (as measured by the pegboard test) was higher in the AGPA group than in the control group. The hand-finger dexterity score of the pegboard test is strongly correlated with cognitive function (Scherder et al., 2008).

A significant risk factor for both stroke and coronary heart disease is hypertension, which is the most important factor for the development of WMHs (Pantoni, 2010). WMHs are associated with an increased risk of stroke (Debette and Markus, 2010; Wardlaw et al., 2013; Debette et al., 2019), cognitive decline (Debette and Markus, 2010; Inaba et al., 2011; Wardlaw et al., 2013; Debette et al., 2019), and functional decline in older age (Poggesi et al., 2011). Older adults who engage in moderate to high levels of physical activity for prolonged periods have smaller WMHs than those who do not exercise (Otsuka et al., 2024). Moreover, physical activity may help to maintain cognitive functions, such as reasoning, processing speed, and vocabulary, even in the presence of a WMH burden (Song et al., 2022). It has been shown that WMHs typically progress over time and do not improve (Schmidt et al., 1999). However, regression of WMH can occur (Moriya et al., 2009; Wardlaw et al., 2017). Our observation of lower WMH grades in individuals who engage in AGPA than those in controls is in line with a previous randomized controlled trial that demonstrated that resistance training slows the progression of WMHs (Bolandzadeh et al., 2015). Furthermore, our logistic regression analysis revealed a fully adjusted odds ratio of 4.88 (1.67–14.28), and a lifestyle without habitual AGPA was significantly associated with a higher prevalence of stroke than a lifestyle with habitual AGPA. Therefore, our findings from both studies collectively suggest that regular AGPA is associated with better markers of neurovascular health, thereby suggesting a potential protective role against neurovascular aging.

It is important to note that the present study and our related publication (Nishiwaki et al., 2025) originate from the same larger research program. Although there is thematic overlap, the two studies address distinct research questions using different cohorts and primary outcomes. The previous study focused on arterial stiffness and dexterity in a community-dwelling cohort, whereas the present manuscript provides a detailed analysis of MRI-defined neuroanatomical markers (i.e., WMHs) and the prevalence of clinical stroke and dementia in a hospital-based cohort. Nonetheless, we have cross-referenced both manuscripts to ensure full transparency.

In conclusion, habitual AGPA may be considered an effective measure against aging-associated brain diseases such as stroke and dementia. However, there is scope for further exploration of the link between AGPA and the development of stroke and dementia. For example, a randomized controlled trial investigating the long-term effects of AGPA on older adults in a larger sample is warranted.

## 5 Strengths and limitations

The experimental component of this study was designed to explore short-term mechanistic responses by measuring changes in key parameters, including arterial stiffness, the fibrinolytic system, and BDNF. Although a sample size of 12 was determined by *a priori* power analysis to detect a medium effect size for our primary outcome of arterial stiffness (see Methods), the statistical power for secondary outcomes, such as cognitive and biomarker changes, may be limited. Additionally, the experimental study included only young men, which markedly limits the generalizability of our findings. The absence of female participants was the result of recruitment challenges at a predominantly male engineering university; no female students responded to our advertisements. This lack of female participants is an important limitation because physiological responses to exercise—particularly in terms of vascular adaptations—can differ between the sexes, partly because of hormonal influences (e.g., estrogen). Future studies must include female participants to determine whether the observed benefits of AGPA extend to women.

Meanwhile, the cross-sectional component of the study examined long-term associations with stroke, dementia, and brain structure in older adults. With regard to the relationship between the two studies, in the case that changes in arterial stiffness and the fibrinolytic system are sustained long term, we consider that it may be possible to prevent the development of white matter lesions in older adults. Moreover, an increase in BDNF and engaging in activities that employ cognitive functions (e.g., executive functions) may prevent cognitive decline in older adults. However, we also recognize that extrapolating acute responses observed in young adults to long-term outcomes in older adult populations must be interpreted with caution. Indeed, acute and chronic adaptations are distinct processes, and thus causal inferences cannot be drawn from our current design. The proposed mechanistic links, although plausible, remain hypothetical and require validation in long-term longitudinal studies. The major strengths of this study are the use of a community-based sample and the highly objective and robust methods used to acquire data. We also conducted several sensitivity analyses to verify the robustness of our results. However, a limitation of the study is potential selection bias because of the relatively modest cohort size. Additionally, although the HDS-R is a validated screening tool for dementia in Japan, it does not provide a comprehensive assessment of specific cognitive domains, such as executive function, memory, or processing speed. Future studies should use a broader battery of neuropsychological tests to better delineate the nuanced effects of AGPA on different facets of cognition. Other potential limitations of the study are that we focused only on older Japanese adults, and that we did not distinguish between occupational and leisure AGPA, which may exert differential effects. Although we assessed the frequency and duration of AGPA in older adults, direct measures of intensity, such as heart rate, metabolic equivalents, and perceived exertion, were not available, which is a notable limitation of our study. It was not possible to obtain more detailed indicators and data from patients via general examinations, medical consultations, or interviews at general medical institutions. However, typically, AGPA corresponds to moderate intensity. Other potential limitations are that the data were from individuals who engaged in either leisure or occupational AGPA, and we focused only on older Japanese adults. Furthermore, our data did not distinguish between occupational and leisure AGPA, which may exert differential effects. We recommend that future studies consider this distinction. One reason for not distinguishing between leisure and occupational AGPA in our study is that our sample included older adults who had originally engaged in occupational AGPA but subsequently transitioned to leisure AGPA as they aged. Although we tested the hypothesis that AGPA induces preventive physiological effects on neurovascular aging, in line with international observational data (Stiernström et al., 2001; Simons et al., 2006; Waggoner et al., 2011; Levêque-Morlais et al., 2015; Buettner and Skemp, 2016), agricultural practices and regional environments may vary considerably among countries. Furthermore, recent epidemiological evidence has shown that although higher leisure-time physical activity is associated with reduced cardiovascular events, including stroke events, higher occupational physical activity is associated with an increased risk of stroke; moreover, these effects are independent of each other (Holtermann et al., 2021). Additionally, the cross-sectional design cannot fully account for all potential confounders. Although our multivariable model adjusted for many clinical variables, residual confounding from unmeasured factors—such as dietary patterns, socioeconomic status, and social engagement—may persist, and might influence both participation in AGPA and neurovascular outcomes. Whether the basis for engaging in AGPA impacts the favorable effects on neurovascular aging requires further research. Nevertheless, to the best of our knowledge, this is the first study to examine the effects of AGPA on surrogate neurovascular aging indices, including stroke and dementia prevalence, using highly objective and robust multiple verification methods. Our results provide a valuable foundation for further large-scale, long-term interventional studies to elucidate the multifaceted preventive effects of AGPA on neurovascular aging.

## 6 Conclusion

The present findings support regular AGPA as a beneficial lifestyle factor that is associated with a lower prevalence of stroke and dementia, potentially through its favorable effects on arterial stiffness, cerebral WMH status, and cognitive function. AGPA offers the combined effects of general physical activity factors and specific AGPA-related factors, and thus may induce similar or even greater benefits than physical activity alone. Regular AGPA may be an effective lifestyle intervention to prevent neurovascular aging, which may have major implications for individual health, as well as the Sustainable Development Goals. In an aging world with urgent ongoing environmental concerns, the promotion of AGPA among individuals may benefit the health of both humans and the environment.

## Data Availability

The raw data supporting the conclusions of this article will be made available by the authors, without undue reservation.
